# Identifying Internet Addiction and Evaluating the Efficacy of Treatment Based on Functional Connectivity Density: A Machine Learning Study

**DOI:** 10.3389/fnins.2021.665578

**Published:** 2021-06-17

**Authors:** Yang Wang, Yun Qin, Hui Li, Dezhong Yao, Bo Sun, Jinnan Gong, Yu Dai, Chao Wen, Lingrui Zhang, Chenchen Zhang, Cheng Luo, Tianmin Zhu

**Affiliations:** ^1^School of Rehabilitation and Health Preservation, Chengdu University of Traditional Chinese Medicine, Chengdu, China; ^2^Key Laboratory for NeuroInformation of Ministry of Education, School of Life Sciences and Technology, University of Electronic Science and Technology of China, Chengdu, China; ^3^School of Medicine, Chengdu University, Chengdu, China; ^4^School of Computer Science, Chengdu University of Information Technology, Chengdu, China; ^5^Department of Chinese Medicine, Chengdu Eighth People’s Hospital, Chengdu, China; ^6^Department of Rehabilitation, Zigong Fifth People’s Hospital, Zigong, China; ^7^Department of Medicine, Leshan Vocational and Technical College, Leshan, China; ^8^Department of Rehabilitation, TCM Hospital of Longquanyi District, Chengdu, China; ^9^Research Unit of NeuroInformation, Chinese Academy of Medical Sciences, Chengdu, China

**Keywords:** internet addiction, cognitive behavior therapy, support vector classification, support vector regression, biomarker

## Abstract

Although mounting neuroimaging studies have greatly improved our understanding of the neurobiological mechanism underlying internet addiction (IA), the results based on traditional group-level comparisons are insufficient in guiding individual clinical practice directly. Specific neuroimaging biomarkers are urgently needed for IA diagnosis and the evaluation of therapy efficacy. Therefore, this study aimed to develop support vector machine (SVM) models to identify IA and assess the efficacy of cognitive behavior therapy (CBT) based on unbiased functional connectivity density (FCD). Resting-state fMRI data were acquired from 27 individuals with IA before and after 8-week CBT sessions and 30 demographically matched healthy controls (HCs). The discriminative FCDs were computed as the features of the support vector classification (SVC) model to identify individuals with IA from HCs, and the changes in these discriminative FCDs after treatment were further used as features of the support vector regression (SVR) model to evaluate the efficacy of CBT. Based on the informative FCDs, our SVC model successfully differentiated individuals with IA from HCs with an accuracy of 82.5% and an area under the curve (AUC) of 0.91. Our SVR model successfully evaluated the efficacy of CBT using the FCD change ratio with a correlation efficient of 0.59. The brain regions contributing to IA classification and CBT efficacy assessment were the left inferior frontal cortex (IFC), middle frontal cortex (MFC) and angular gyrus (AG), the right premotor cortex (PMC) and middle cingulate cortex (MCC), and the bilateral cerebellum, orbitofrontal cortex (OFC) and superior frontal cortex (SFC). These findings confirmed the FCDs of hyperactive impulsive habit system, hypoactive reflecting system and sensitive interoceptive reward awareness system as potential neuroimaging biomarkers for IA, which might provide objective indexes for the diagnosis and efficacy evaluation of IA.

## Introduction

In the last two decades, with the development of digital information technology, the internet has brought great convenience and benefit to people’s lives, but the popularity of the internet has also brought new public health issues, such as internet addiction (IA). IA, characterized by excessive internet craving and habitual and uncontrolled use of the internet (Hormes et al., 2014; Wang et al., 2019), has widely prevailed all over the world, with a prevalence ranging from 0.2 to 57.5% (Darvesh et al., 2020). Even in medical students, the pooled incidence rate of IA is as high as 30.1% (Zhang et al., 2018). Furthermore, the incidence of IA is rising rapidly. The prevalence rate of IA has risen steeply almost ninefold in Hong Kong in the last 10 years (Chung et al., 2019). Additionally, with the popularity of mobile internet, the risk of IA in children has become an increasing concern (Mihajlov and Vejmelka, 2017). Surveys show that almost one-quarter of early teenagers spend 40 h online per week (Ayar et al., 2017), and more than 30% of children under 2 years old have used mobile internet devices (Young, 2017), reflecting the younger age trend of IA. Collectively, epidemiological features, including worldwide prevalence, high incidence, rapidly increasing incidence and younger age trend, make IA a public health threat as serious as substance addiction.

As one of the most widely used noninvasive technologies for investigating brain function *in vivo*, functional magnetic resonance imaging (fMRI) has greatly improved our understanding of the neuropathological abnormalities underlying IA. Using resting-state functional connectivity (FC), an fMRI technology reflecting the functional communication of preselected regions of interest (ROIs), researchers have found hyperactive function of the striatum and orbitofrontal cortex (OFC) (Kuehn and Gallinat, 2015; Ge et al., 2017), indicating that IA has similar pathological reward awareness to substance addiction. In a longitudinal fMRI study, the FC of the putamen in individuals with IA was also found to be significantly correlated with the online time per day, suggesting that the habitual use of the internet was related to hyperactive impulsive habit system in the brain (Lee et al., 2021). Using functional connectivity density (FCD) analysis, a novel method that overcomes the bias of FC produced by the preselection of ROIs, to analyze whole-brain functional communication, we further found altered dorsolateral prefrontal cortex (DLPFC) function in our prior study (Wang et al., 2019), representing the defective reflecting system in IA. These fMRI studies have provided us with the potential neuropathologic mechanism underlying IA; however, the average differences between groups are insufficient in guiding individual clinical practice directly. There is still a lack of biomarkers to diagnose IA and evaluate the effectiveness of therapy on IA.

Recently, machine learning (ML) has increasingly gained popularity in the neuroimaging research field. Through appropriate features, ML models can identify neuropsychiatric diseases and predict the effectiveness of treatment accurately, which provides us with an available way to explore potential biomarkers for neuropsychosis diagnosis and the evaluation of therapy efficacy (Arbabshirani et al., 2017). Feature selection, aiming to find appropriate features to develop model, is a necessary step in ML studies, especially in the neuroimaging field. Due to the vast amount of data in neuroimaging studies, overfitting is inevitable without feature selection (Chiang et al., 2015). The two-sample *t*-test is a commonly used approach for feature selection in pattern recognition, which can determine the features distributed differently in two groups, meaning that the corresponding features have excellent discrimination ability (Liu et al., 2019). Support vector machine (SVM), which includes support vector classification (SVC) and supporter vector regression (SVR), is currently the most popular algorithm applied in neuroimaging studies for its outstanding performance on pattern recognition and regression prediction in small-sample datasets (Bruin et al., 2019). Using the FC of the ventral tegmental area and substantia nigra to build an SVC model, Wen et al. (2020) successfully identified individuals with IA, implicating the potential of the functional connectivity indexes of interoceptive reward awareness system as neuroimaging markers of IA. However, due to the priori selection of ROIs, seed-based FC can only partly provide SVC with local features referred to ROIs. The lack of global features of information communication might reduce the performance of SVC (Guo et al., 2017). Additionally, no studies have focused on the evaluation of IA treatment efficacy, although SVR has been applied in the evaluation of IA severity (Song et al., 2020). Thus, an SVM study based on more comprehensive features is needed to explore potential effective biomarkers for both IA diagnosis and the evaluation of therapy efficacy.

Functional connectivity density analysis, computing the temporal correlations of each voxel to all other voxels, can provide SVM with more comprehensive features without bias. Based on voxel-based FCD, data-driven SVM with global features will provide us with an opportunity to acquire specific neuroimaging markers for IA diagnosis and the evaluation of therapy efficacy. Thus, this study aims to explore potential biomarkers for IA diagnosis and the evaluation of therapy efficacy using a combination of FCD analysis and SVM models. For this purpose, we first computed the local FCD (lFCD), long-range FCD (lrFCD), and global FCD (gFCD) of 27 individuals with IA and 30 healthy controls (HCs) and then selected the discriminative FCDs as effective features by a 2-sample *t*-test. After parameter optimization, SVC models were established to identify IA. After cognitive behavior therapy (CBT), an effective treatment for various addictions, including IA (Goslarp et al., 2020), FCD changes were used as features in SVR for the evaluation of the efficacy of CBT. We hypothesized that (1) FCDs would accurately discriminate individuals with IA from HCs by the SVC model, and (2) FCD changes would well represent symptom improvement after CBT by the SVR model.

## Materials and Methods

### Participants

The study protocol was approved by the Sichuan Regional Ethics Review Committee on Traditional Chinese Medicine (2016KL-005) and carried out in accordance with the Declaration of Helsinki. All enrolled participants voluntarily participated in the study and signed informed consent forms before inclusion.

Sixty participants (30 individuals with IA and 30 HCs) aged between 18 and 30 were initially recruited from universities. Three individuals with IA failed to complete the CBT sessions due to scheduling conflicts or personal issues. Thus, the final dataset included data obtained from 27 individuals with IA and 30 HCs. Fifteen of 27 individuals with IA had participated in our former fMRI study detecting functional abnormalities in IA (Wang et al., 2019) and further consented to take part in the present CBT study. Each individual with IA met the diagnostic criteria for IA (assessed by the Internet Addiction Questionnaire), while all the demographically matched HCs did not meet the criteria for IA. The Internet Addiction Questionnaire developed by Young was the first diagnostic tool for IA (Young, 1998). Based on the original version of Young, Beard proposed a wider applied questionnaire (Beard and Wolf, 2001), which was composed of eight items. The first five items were characteristics of IA, and the other three were negative consequences of IA. All of the first five items and at least one of the last three items were required for a diagnosis of IA. Young’s Internet Addiction Test (IAT) was used to assess the severity of IA, and a score greater than 50 was required in the IA group (Yoon et al., 2017). Participants with organic diseases or a history of substance abuse (such as alcohol dependence, nicotine addiction or any other drug addictions) were excluded from our study. Additionally, we also excluded pregnant or lactating females.

### Questionnaire

In addition to IAT, the Self-rating Depression Scale (SDS) and Self-rating Anxiety Scale (SAS) were completed by all participants and were applied to assess depression and anxiety, respectively. All these clinical scales were translated into Chinese versions. We also collected information on sex, age, and years of education by a self-designed questionnaire.

### Cognitive Behavior Therapy

**FIGURE 1:**
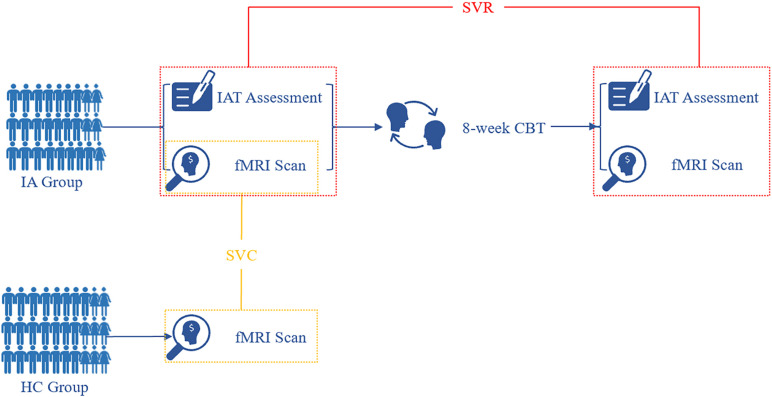
Flow diagram of study procedure. At baseline, fMRI images were acquired from both the HC and IA groups. Based on informative FCDs, the SVC model was applied to the identification of individuals with IA. After 8-week CBT sessions, individuals with IA were rescanned for fMRI data. The change in informative FCDs was used to evaluate symptom improvement by the SVR model. IA, internet addiction; HC, heathy control; SVC, support vector classification; SVR, support vector regression; IAT, Young’s Internet Addiction Test; CBT, cognitive behavior therapy.

The complete procedure of this study is shown in Figure 1. After baseline assessment and fMRI scan, all individuals with IA were treated with CBT, which was led by an experienced licensed psychotherapist. A total of 27 individuals with IA completed the 8-week CBT protocol. The CBT protocol consisted of 8 sessions, and each session lasted 1.5–2 h. According to the modularized CBT protocol slightly modified from Park’s version (Park et al., 2016), the following topics were discussed in the 8 sessions: (1) an introduction of the negative consequences of excessive use of the internet, (2) the motivation behind excessive internet use, (3) techniques for managing pressure, (4) techniques for recognizing addiction when it happens, (5) five steps to change, (6) techniques to deal with problems, (7) techniques to recover family relationships; and (8) future plans. One topic was discussed in each session.

To ensure the pure effect of CBT, individuals with IA were informed not to take any other medicines or interventions during the research term. Within 3 days after CBT sessions, we assessed symptom severity again by IAT.

### Resting-State fMRI Data Acquisition

All fMRI images were acquired by a 3.0 T MR imaging system (GE Discovery MR 750, United States) with a standard 8-channel head coil. The HC group took part in only one fMRI scan, while the IA group participated in two scans, one within 3 days before CBT treatment and the other within 3 days after CBT sessions. During data collection, ear plugs and soft pads were used to restrict noise and displacement of head, respectively. The participants were instructed to keep their eyes closed and remain awake with nothing in mind during the whole scanning term. A standard echo planar imaging sequence was adopted to collect functional images, with the following parameters in our prior study (Wang et al., 2020a): repetition time = 2000 ms, echo time = 30 ms, flip angle = 90°, field of view = 24 cm × 24 cm, image matrix = 64 × 64, no gap, and voxel size = 3.75 mm × 3.75 mm × 4.4 mm. For this scan sequence, 255 volumes were obtained, and each volume included thirty-five transverse slices.

### Date Preprocessing and FCD Calculation

The Neuroscience Information Toolbox (NIT)^1^ was used for data preprocessing. To minimize the influence of an unstable magnetic field in the initial scanning, the first 5 volumes of each participant were discarded. Subsequently, slice timing and spatial realignment were conducted to correct time delay and head motion, respectively. Participants with more than 2° rotation or more than 2 mm displacement were excluded from the present study. The functional images were then spatially normalized to a standard Montreal Neurological Institute (MNI) template and resampled to 3 mm × 3 mm × 3 mm. After that, we regressed out nuisance signals, including 24 head motion parameters and signals from cerebral spinal fluid and white matter. Ultimately, to reduce the interference of low-frequency drift and high-frequency noise, bandpass filtering (0.01–0.08 Hz) was conducted.

To address concerns about the influence of head motion on fMRI analysis, voxel-specific framewise displacement (FD) was computed (Jenkinson et al., 2002; Power et al., 2012; Yan et al., 2013). Two-sample *t*-test showed that there was no significant intergroup difference at baseline (*P* > 0.05, mean ± SD: 0.0407 ± 0.0123 for HCs and 0.0419 ± 0.0172 for the IA group). Additionally, no significant difference before and after CBT in the IA group was observed based on the paired *t*-test (*P* > 0.05, mean ± SD: 0.0419 ± 0.0172 for pretreatment and 0.0436 ± 0.0189 for posttreatment). Furthermore, to rule out the effect of head motion on the following analyses, we also calculated FCD with and without scrubbing the frames with FD > 0.5 (Yamashita et al., 2020).

In our study, the FCD was calculated using the NIT according to the method proposed by Tomasi and Volkow (2010a, b). Initially, Pearson correlations between the time course of each voxel and those of other voxels were calculated. The connections between two voxels with a correlation coefficient of *R* > 0.6 were considered as significant connections according to the previous study (Tomasi and Volkow, 2010a). The gFCD was defined as the total number of efficient functional connections between a given voxel and all other voxels. The lFCD was defined as the total number of efficient functional connections between a given voxel and its neighboring voxels. The lrFCD was defined as the number of efficient functional connections between a given voxel and other distant voxels. Thus, the combination of gFCD, lFCD, and lrFCD can well describe the role of a given voxel hub in global, local and long-range information transmission. Finally, the whole-brain gFCD, lFCD, and lrFCD maps were spatially smoothed with a Gaussian kernel of 6 mm.

### SVM Modeling

**FIGURE 2:**
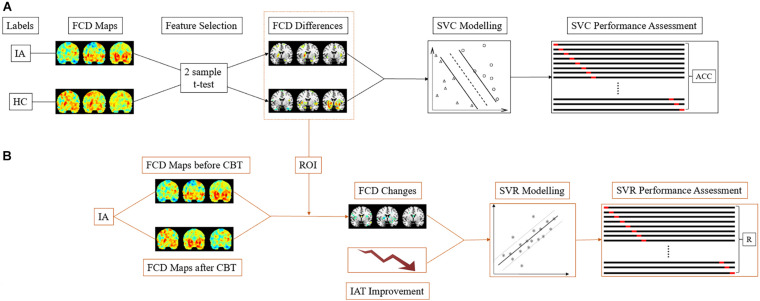
The SVM schematic flow. **(A)** In the SVC model, the gFCD, lFCD, and lrFCD differences between the HC and IA groups (pretreatment) were used as features for pattern identification. Leave-one-out cross-validation was applied to performance assessment. **(B)** In the SVR model, the alterations of discriminative gFCD, lFCD, and lrFCD were used as features to predict the improvement of IAT. The performance of the SVR model was represented by the correlation of actual IAT scores to predict IAT scores collected in each leave-one-out-cross-validation fold. IA, internet addiction; HC, heathy control; SVC, support vector classification; SVR, support vector regression; IAT, Young’s Internet Addiction Test; CBT, cognitive behavior therapy; ACC, accuracy; R, correlation coefficient.

The main steps of SVM modeling are described in Figure 2, comprising feature selection, model building, and performance evaluation (Jie et al., 2018; Wang et al., 2020b). Due to the vast amount of data, the selection of informative features from tremendous neuroimaging data is necessary in ML studies that are based on neuroimaging (Chiang et al., 2015) since it decreases the computational burden and improves the performance of SVM models. As in previous study, the two-sample *t*-test (*p* < 0.005) was used to select features in the current study (Suk et al., 2015; Kwak et al., 2020). After the features with discriminative information were selected, we adopted the Fisher score as the feature weight in the SVC model (Germond et al., 2018), which is defined in the following equation:

$$F\,S\,\left(i\right)=\frac{n_{1}\,{\left(m_{1\,i}-m_{i}\right)}^{2}+n_{2}\,{\left(m_{2\,i}-m_{i}\right)}^{2}}{n_{1}\,\sigma_{1\,i}^{2}+n_{2}\,\sigma_{2\,i}^{2}}$$

Here, m_*i*_ represents the average of the i-th feature in all samples, n_1_ and n_2_ are the number of samples in the HC and IA groups, m_1i_ and m_2i_ are the respective mean values of the i-th feature in each group, and $\sigma_{1\,i}^{2}$ and $\sigma_{2\,i}^{2}$ represent the respective variance of the i-th feature in each group. Since the Fisher score ranks features in order of how discriminate they are, consequently, a higher Fisher score value contributes more information to the SVC model.

To further confirm the selected FCDs as potential biomarkers, the ratio of changes in the informative FCDs in IA group was introduced into the SVR model as features to assess the efficacy of CBT. The contribution of each feature to the SVR model was evaluated by mutual information, which weighs the features by the correlation and redundancy of features. High mutual information represents a high prediction ability and low redundancy (Gan et al., 2019).

LIBSVM^2^ was implemented in this study to build SVC and SVR models (Chang and Lin, 2011). For classification, the binary SVC model with a radial basis function (RBF) kernel was built according to the extracted discriminative FCDs of the IA (pretreatment) and HC groups. To optimize the classifier and evaluate the performance of SVC, we used leave-one-out cross-validation (LOOCV) and grid search (hyperparameter optimization for C and gamma) to train the data. The performance of the classification was described as the mean accuracy in LOOCV and area under the curve (AUC). In addition, a permutation test was applied with 1000 rounds to assess the significance of classification accuracy (*P* < 0.05).

Through building the SVR model, we aimed to evaluate symptom improvement by FCD changes after CBT in individuals with IA. Thus, the ratio of FCD changes (change/baseline of FCD) and IAT score improvement (change/baseline of IAT score) were introduced into the SVR model as features and labels, respectively. Grid search and LOOCV were also used to optimize and assess the performance of SVR. Pearson’s correlation coefficient was computed between the actual IAT scores and the predictive scores collected in all LOOCV folds. A 1000 times permutation test with a P-value < 0.05 was further performed to ensure the significance of the result.

### Statistical Analysis

Demographic characteristics and treatment response were analyzed by SPSS 18. Continuous variable (e.g., age, IAT score) differences between HCs and individuals with IA at baseline were compared by two sample *t*-test, while the difference within the IA group before and after CBT was compared by paired *t*-test. Categorical variable (e.g., gender) differences were compared by the chi-squared test.

As described in section “SVM Modeling,” the findings of two-sample *t*-test were applied to feature selection using the Resting-State fMRI Data Analysis Toolkit plus (RESTplus V1.24)^3^ (Jia et al., 2019).

## Results

### Demographic Characteristics and Treatment Response

A total of 57 participants (27 individuals with IA and 30 HCs) were included in the final data analysis. No statistically significant differences were observed in sex, age, or years of education between the IA group and the HC group (*P* > 0.05). Individuals with IA exhibited higher IAT, SDS, and SAS scores (*P* < 0.05). After 8 weeks of CBT, significant decreases in IAT, SDS, and SAS scores were observed in the IA group (*P* < 0.05) (see Table 1).

**TABLE 1:** Demographic characteristics and treatment response.

	**Healthy control group (*n* = 30)**	**IA group pretreatment (*n* = 27)**	**IA group post treatment (*n* = 27)**
	**M ± SD**	**M ± SD**	**M ± SD**
Age (years)	21.73 ± 2.08	20.74 ± 1.95^#^	20.74 ± 1.95
Sex (male/female)	22/8	22/5^#^	22/5
Education (years)	15.77 ± 1.82	14.70 ± 1.84^#^	14.70 ± 1.84
Internet Addiction Test Scale	29.90 ± 7.18	62.89 ± 11.57^▲^	46.56 ± 11.83*
Self-rating Depression Scale	37.70 ± 7.87	55.74 ± 9.09^▲^	50.22 ± 10.28*
Self-rating Anxiety Scale	35.00 ± 6.79	50.11 ± 11.05^▲^	44.22 ± 9.61*

*^#^Comparison between individuals with IA and HCs at baseline, *P* > 0.05.*

^▲^Comparison between individuals with IA and HCs at baseline, *P* < 0.05.

*Comparison in the IA group before and after CBT, *P* < 0.05.

### Feature Selection

**FIGURE 3:**
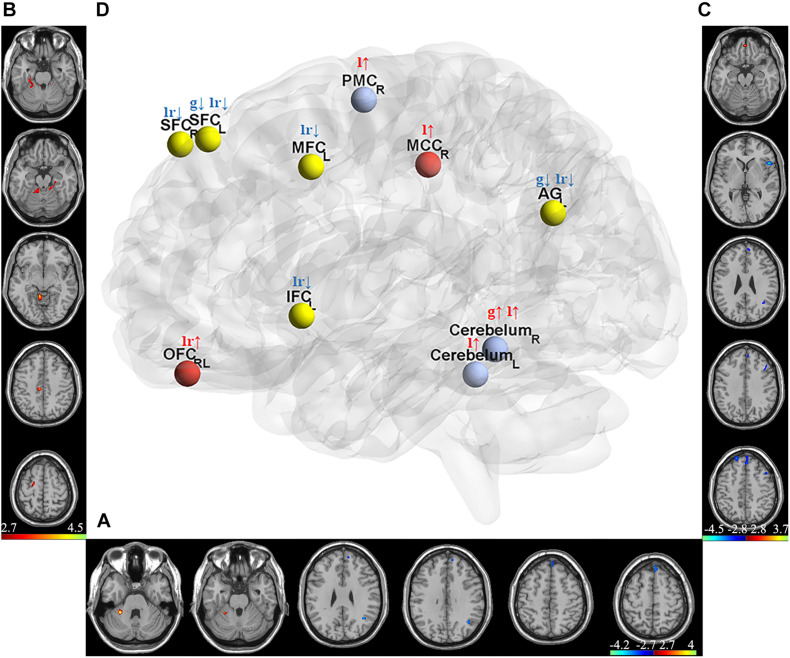
Discriminative gFCD, lFCD, and lrFCD between HCs and individuals with IA**. (A)** Discriminative gFCD. **(B)** Discriminative lFCD. **(C)** Discriminative lrFCD and **(D)** The summary of these discriminative FCDs based on BrainNet Viewer (http://www.nitrc.org/projects/bnv/) (Xia et al., 2013). The red nodes represent the interoceptive reward awareness system, the blue nodes represent the impulsive habit system, and the yellow nodes represent the reflecting system. The red up arrows represent increased FCDs, while the blue down arrows represent decreased FCDs. g, gFCD; l, lFCD; lr, lrFCD; L, left; R, right; SFC, superior frontal cortex; OFC, orbitofrontal cortex; MFC, middle frontal cortex; IFC, inferior frontal cortex; PMC, premotor cortex; MCC, middle cingulate cortex; AG, angular gyrus.

Two-sample *t*-test was used to select informative features for subsequent model building. The indiscriminative FCDs were filtered out, and only FCDs with a P-value < 0.005 were selected as feature vectors (see Table 2). As shown in Figure 3A, in comparison to HCs, individuals with IA exhibited increased gFCD in the right cerebellum and decreased gFCD in the left angular gyrus (AG) and superior frontal cortex (SFC). Figure 3B shows the enhanced lFCD of individuals with IA in the right PMC, middle cingulate cortex (MCC), and the bilateral cerebellum. The IA group also showed higher lrFCD in the left/right orbitofrontal cortex (OFC) as well as lower lrFCD in the left AG, inferior frontal cortex (IFC), and middle frontal cortex (MFC) and the bilateral SFC, as shown in Figure 3C.

**TABLE 2:** Discriminative gFCD, lFCD and lrFCD between HCs and individuals with IA.

**FCDs**	**Brain**	**Cluster**	**MNI**			**Peak**	***P* value**
	**regions**	**voxels**	**coordinates**			***T*-value**	
			**X**	**Y**	**Z**		
gFCD	Cerebellum_R	26	28	−46	−30	3.9005	< 0.005
	AG_L	29	−39	−57	27	–3.6592	<0.005
	SFC_L	98	−6	33	57	–3.6342	<0.005
lFCD	Cerebellum_R	129	9	−48	−15	4.3797	<0.005
	MCC_R	23	6	−27	42	3.6251	<0.005
	PMC_R	31	21	−9	61	3.5222	<0.005
	Cerebellum_L	36	−15	−39	−21	3.2990	<0.005
lrFCD	OFC_L+R	43	0	48	−21	3.3400	<0.005
	IFC_L	25	−51	19	0	–4.3605	<0.005
	SFC_L	125	−3	42	51	–4.0600	<0.005
	SFC_R	24	18	48	48	–3.8860	<0.005
	AG_L	28	−39	−60	30	–3.7300	<0.005
	MFC_L	46	−42	15	45	–3.5797	<0.005

*MNI, Montreal Neurological Institute; L, left; R, right; PMC, premotor cortex; IFC, inferior frontal cortex; SFC, superior frontal cortex; AG, angular gyrus; MCC = middle cingulate cortex; OFC, orbitofrontal cortex; MFC, middle frontal cortex.*

According to the tripartite neurocognitive model (Wei et al., 2017), these results can be summarized as hyperactive impulsive habit system, hypoactive reflecting system, and sensitive interoceptive awareness system, which were proposed as the main pathological mechanism of IA (Figure 3D). With all bad frames scrubbed, the reanalysis produced similar results, which are shown in Supplementary Figure 1.

### SVC Results

**FIGURE 4:**
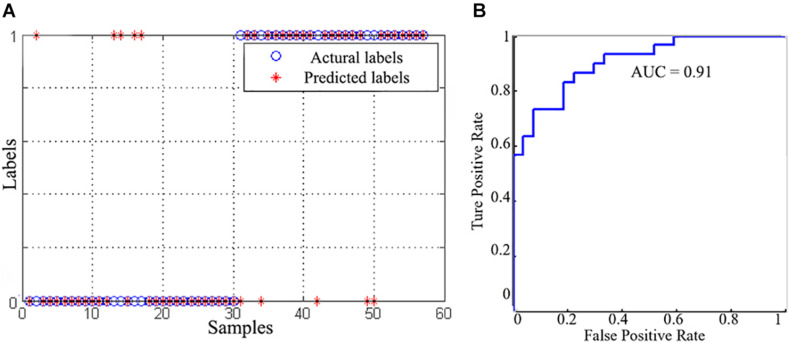
The classification performance of SVC model. **(A)** The classification results between the IA and HC groups. **(B)** The receiver operating characteristic curve of the SVC model.

Using gFCD, lFCD and lrFCD, the binary SVC successfully discriminated individuals with IA from HCs with a mean accuracy of 82.5% (Figure 4A), which was ensured by a permutation test (*P* < 0.05). Figure 4B shows the receiver operating characteristic (ROC) curve of this SVC model, the AUC of which is 0.91. Similar results were also obtained after scrubbing the bad frames (Supplementary Figure 2).

### SVR Results

**FIGURE 5:**
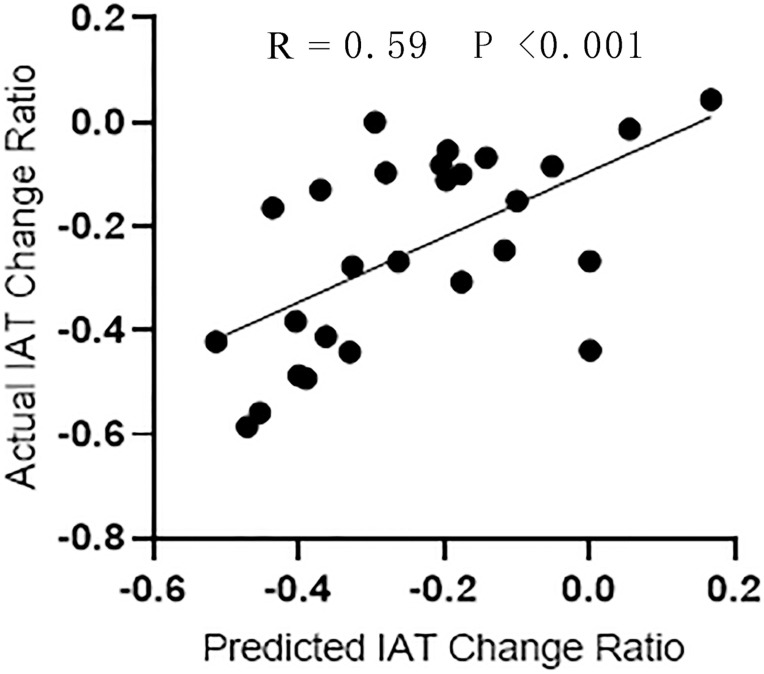
The regression performance of SVR model. Correlation analysis between the actual IAT change ratio and the predictive change ratio predicted by SVR.

As shown in Figure 5, the SVR model successfully predicted symptom improvement after CBT. Based on the variation ratio of FCDs (change/baseline), the SVR model predicted an IAT score decrease after treatment with a correlation efficient of 0.59. The results were validated by a permutation test (*P* < 0.05). The repeated analysis after the bad frames were scrubbed produced similar results (Supplementary Figure 3).

### Feature Contribution

**FIGURE 6:**
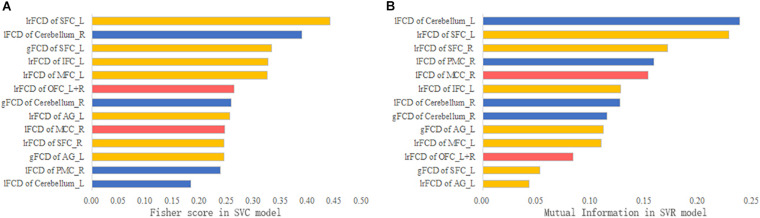
The feature contribution. **(A)** The Fisher score of each feature in the SVC model. **(B)** The mutual information of each feature in the SVR model.

The contribution of each feature to the SVM models is ranked in Figure 6. According to the Fisher scores, the lFCD of the right cerebellum and the lrFCD and gFCD of the left SFC were the most important features in the SVC model (Figure 6A). The lFCD of the left cerebellum and the lrFCD of the bilateral SFC contributed most to the SVR model, since those features had the highest mutual information (Figure 6B).

## Discussion

Using data-driven SVM models, we successfully identified individuals with IA and evaluated the effectiveness of CBT. To our knowledge, the present study is the first to explore unified biomarkers for both diagnosis and therapy efficacy evaluation in psychological neuroimaging using SVM models. The brain regions contributing to IA classification and the evaluation of CBT efficacy were the left IFC, MFC and AG, the right PMC and MCC, and the bilateral SFC, OFC and cerebellum, which were compatible with the tripartite neurocognitive model of IA (Wei et al., 2017). In the well-accepted tripartite neurocognitive model, the pathological mechanism of IA was attributed to three key abnormal systems — hyperactive impulsive habit system, hypoactive reflecting system, and sensitive interoceptive awareness system (Wei et al., 2017).

### Hyperactive Impulsive Habit System

The impulsive habit system in the tripartite neurocognitive model is responsible for fast, automatic, unconscious, and habitual behaviors (Wei et al., 2017). In the current study, the hyperfunction of the right PMC and bilateral cerebellum in individuals with IA, which were thought to contribute to the habitual or compulsive use of the internet, was detected and further found to contribute to IA identification and the evaluation of CBT efficacy (Wang et al., 2020a).

As a key neural system that generates automatized behavior to adapt to circumstances, the habit system is dedicated to transiting goal-directed behavior to response-stimulate habitual behavior (Everitt and Robbins, 2005). The hyperactive habit system is considered part of the mechanism underlying substance dependence (Zilverstand et al., 2018), accounting for the transition from voluntary drug use (response-outcome goal-directed behavior) to habitual and compulsive drug abuse (response-stimulate habitual behavior) (Everitt and Robbins, 2005). As a behavioral addiction, IA manifests with habitual and compulsive use of the internet (Seo and Ray, 2019). Under the framework of associative learning theory, previous psychological studies have revealed the aberrant transition process from goal-directed behavior to habit-based behavior in individuals with IA (Zhou et al., 2018; Gong et al., 2020), demonstrating the abnormal habit behavioral pattern built by continuous online playing.

Neuroimaging evidence of aberrant habit system in IA was also revealed by neuropathology studies. A number of studies have found altered function of the PMC in IA (Cheng and Liu, 2020; Shin et al., 2020), which is a key region of the habit system accounting for the transformation of memory into a particular response sequence (Abe and Hanakawa, 2009). The cerebellum, traditionally considered a pure motor center, is now concerned for its various cognitive functions, including habit formation. The cerebellum is involved in rapid and automatic behavioral responses in substance dependence, accounting for the habitual and compulsive drug abuse (Miquel et al., 2019, 2020). In IA studies, increased activations in the cerebellum were also found in individuals with IA when exposed to internet cues, implicating automatic habitual reactions to internet (Schmitgen et al., 2020). Furthermore, the hyperactivity of the PMC-cerebellar loop was found both in IA and substance addiction (Yalachkov et al., 2009; Wang et al., 2020a), suggesting the common pathological changes in the habit system in IA and substance dependence.

In line with the tripartite neurocognitive model, the hyperactive impulsive habit system was found valuable in IA identification and the evaluation of therapy efficacy in our study, further clarifying the abnormal habit formation process in habitual and compulsive use of the internet. According to the Fisher scores, the right cerebellum was one of the most contributive regions to the SVC model, indicating that the hyperconnected right cerebellum might be the discriminative characteristic of IA. The mutual information further revealed that the reduced connectivity of the left cerebellum contributed most to the SVR model, implicating that the effect of CBT on IA was closely associated with the compensatory decrease in left cerebellum connectivity. Thus, the impulsive habit system might be the key factor for IA diagnosis and treatment.

### Hypoactive Reflecting System

According to the tripartite neurocognitive model, the reflecting system is involved in planning, problem solving, and inhibition control (Wei et al., 2017). In the present study, hypoactive FCDs in the right DLPFC (comprising the SFC and MFC) and the left AG, IFC and DLPFC were revealed and further demonstrated to be conducive to IA identification and treatment efficacy prediction. All these regions were included in the reflecting system in the tripartite neurocognitive model.

Containing the most cognitive structures in the brain, the reflecting system is involved in a variety of advanced cognitive functions, including inhibition and problem solving, so it is also known as cognitive control system in other studies. The ability to suppress inappropriate behavior is a main function of the reflecting system, the impairment of which was proposed as a key determinant for uncontrolled internet and drug use (Volkow et al., 2010; Brand et al., 2019). The reflecting system in IA and substance addiction was consistently found to be dysfunctional when performing inhibition control tasks (Darnai et al., 2019; Antons and Matthias, 2020; Suarez-Suarez et al., 2020); therefore, it was regarded as a therapeutic target for addiction. After noninvasive treatment targeted to the system, the addiction symptoms were significantly relieved (Newman-Norlund et al., 2020; Su et al., 2020), indicating the critical role of the reflective system in addiction.

The IFC and DLPFC, responsible for inhibiting response habits and impulsive behavior, are acknowledged as key components of the reflecting system. Impairments in the IFC and DLPFC are thought to be directly related to uncontrolled drug abuse (Ely et al., 2020; Qian et al., 2020), and the stimulation of these regions was demonstrated to be effective in relieving addictive symptoms (Chen et al., 2020b; Newman-Norlund et al., 2020). In IA, a close association between the dysfunction of IFC and DLPFC and the uncontrolled online playing has also been demonstrated (Dong et al., 2020). Although the AG is not typically associated with executive control function, the crucial role of the AG in inhibition control has been well demonstrated by previous studies (Castelluccio et al., 2014; Nobusako et al., 2017; Lewis et al., 2019). In substance dependence research, dysfunction of AG in response inhibition tasks were found to be related to addiction severity (Herman et al., 2019) and could even predict potential substance abuse in the future (Mahmood et al., 2013), demonstrating the importance of AG in inhibiting uncontrolled addictive behavior. In individuals with IA, dysfunctional AG was also a key characteristic change correlating to symptom severity (Lemenager et al., 2016; Chen et al., 2020a), implicating the defective inhibition control function in IA.

In the tripartite neurocognitive model, the reflecting system was described as the controller of the impulsive habit system (Wei et al., 2017). Hence, it is not surprising that the hypoactive reflecting system in our study contributed to IA diagnosis and evaluation of therapy effectiveness. Through Fisher score ranking, the critical role of the DLPFC in IA diagnosis was well demonstrated in the SVC model. Furthermore, the key contribution of the DLPFC to CBT efficacy assessment was revealed by mutual information ranking. Thus, the hypoconnection of the reflecting system might be the main characteristic of IA, and activation connectivity of the reflecting system might be the main effect of CBT on IA.

### Sensitive Interoceptive Awareness System

According to the tripartite neurocognitive model, the sensitive interoceptive awareness system potentiates the activity of the impulsive habit system and undermines the activity of the reflecting system, thus playing an important role in developing and maintaining IA (Wei et al., 2017). Reward awareness plays an important part in interoceptive awareness. In our study, the well-known reward regions OFC and MCC were demonstrated to contribute to IA diagnosis and treatment efficacy evaluation, validating the critical role of sensitive reward awareness in IA.

Comprising of two major dopamine pathways (mesolimbic and mesocortical pathways) in the brain, the reward network plays a decisive role in substance addiction. Previous research has revealed that independent of the drugs used and the tasks performed, the hyperactive reward network was consistently related to craving, addiction severity, and the use duration and frequency of drug use. Consequently, the dysfunction of the reward network was proposed as a fundamental pathological change in substance dependence (Zilverstand et al., 2018). Similar to that in substance addiction, sensitive reward awareness was also demonstrated in IA and thought to be a critical pathological characteristic of IA (Li et al., 2015). The mesocortical dopamine pathway, including the MCC and OFC, is responsible for the cognitive component of reward processing (Leroy et al., 2012). The dysfunction of these two regions was thought to be involved in excessive craving for addictive substances. Analogously, the abnormal microstructure and function of the MCC and OFC have also been found to be closely associated with internet craving (Ko et al., 2009; Lee et al., 2017, 2019), implicating the common foundation of IA and substance dependence.

Compatible with the tripartite neurocognitive model, the sensitive interoceptive awareness system was found in individuals with IA in the present study. In addition, similar to a previous study, the functional connectivity indexes of the reward awareness system were successfully applied to IA classification, verifying the reward awareness system as the key characteristic of IA (Wen et al., 2020). Moreover, we successfully evaluated the effectiveness of CBT using FCD changes in the reward awareness system, further confirming the potential of sensitive reward system as biomarkers of IA.

## Conclusion

In summary, based on individual FCD data, this study successfully differentiated individuals with IA from HCs and further evaluated the effectiveness of CBT. These findings suggested the FCDs of hyperactive impulsive habit system, hypoactive reflecting system, and sensitive interoceptive awareness system as potential neuroimaging biomarkers of IA, providing objective indexes for the diagnosis of IA and the evaluation of treatment efficacy.

## Limitations

Although providing robust and explainable results, our study still has several limitations to be noted. First, to include more participants, we included all types of IA in this study. Further research on specific IA subtypes (such as internet gaming disorder) should be conducted in the future. Second, the clinical endpoint was set at the end of treatment in the present study but should be extended to explore the long-term effect of CBT and study the reduction in online time/week by CBT. Third, due to the limited sample size, these findings need to be validated in other larger datasets. In addition, our SVM models were built on discriminative features. The excluded brain areas may also contain valuable information, which should be considered in future studies. Last, multimodal neuroimaging data (such as structural data) may provide complementary information; thus, multiple modalities are needed to improve the performance of models.

## Data Availability Statement

The raw data supporting the conclusions of this article will be made available by the authors, without undue reservation.

## Ethics Statement

The studies involving human participants were reviewed and approved by Sichuan Regional Ethics Review Committee on Traditional Chinese Medicine. The patients/participants provided their written informed consent to participate in this study.

## Author Contributions

TZ, CL, DY, and YW: conceptualization and methodology. TZ and CL: funding acquisition. HL: supervision. YD, CW, LZ, and CZ: investigation. YQ, BS, and YW: formal analysis. JG: software. YW: writing – original draft. CL: writing – review and editing. All authors contributed to the article and approved the submitted version.

## Conflict of Interest

The authors declare that the research was conducted in the absence of any commercial or financial relationships that could be construed as a potential conflict of interest.

## Footnotes

1 http://www.neuro.uestc.edu.cn/NIT.html

2 https://www.csie.ntu.edu.tw/~cjlin/libsvm/

3 http://www.restfmri.net/forum/restplus

## Abbreviations

AG: angular gyrus
AUC: area under curve
CBT: cognitive behavior therapy
DLPFC: dorsolateral prefrontal cortex
FC: functional connectivity
FCD: functional connectivity density
fMRI: functional magnetic resonance imaging
HC: healthy control
IA: internet addiction
IAT: Young’s Internet Addiction Test
IFC: inferior frontal cortex
LOOCV: leave-one-out-cross-validation
MCC: middle cingulate cortex
MFC: middle frontal cortex
ML: machine learning
OFC: orbitofrontal cortex
PMC: premotor cortex
RBF: radial basis function
ROC: receiver operating characteristic
ROI: regions of interest
SFC: superior frontal cortex
SVC: support vector classification
SVM: support vector machine
SVR: support vector regression.
