# Supplementary material for: Identifying Internet Addiction and Evaluating the Efficacy of Treatment Based on Functional Connectivity Density: A Machine Learning Study
Source: Front Neurosci. 2021 Jun 17;15:665578. doi: 10.3389/fnins.2021.665578 (PMC8247769; doi:10.3389/fnins.2021.665578)
Supplement: Supplementary file 1 [file Table_1.docx]

Supplementary Material


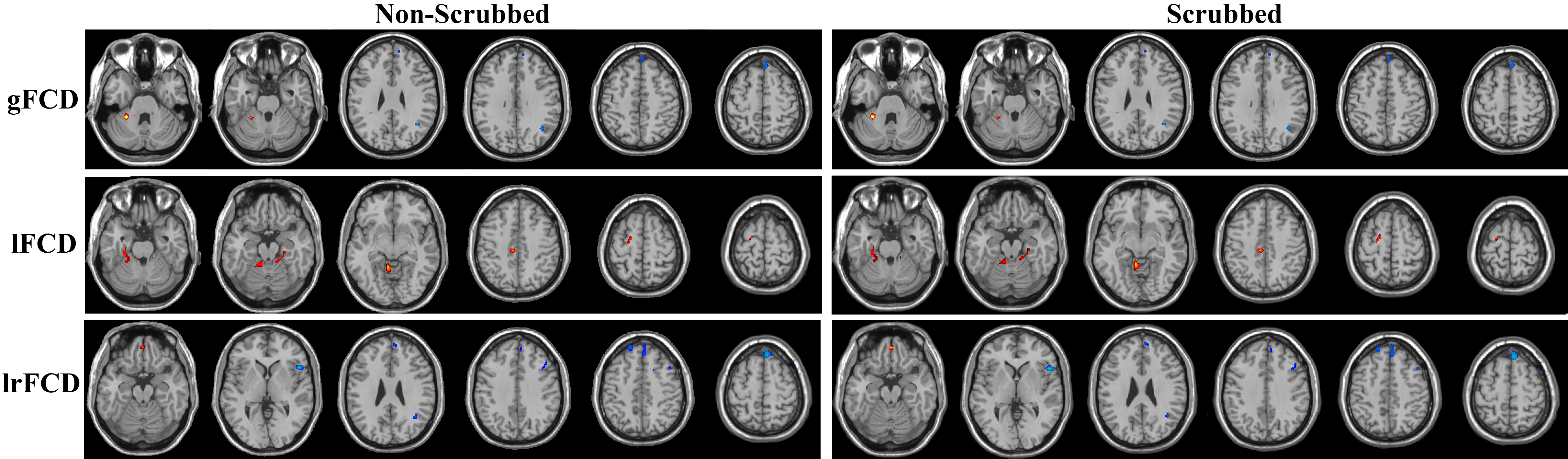


**Supplementary Figure 1. The discriminative gFCD, lFCD and lrFCD analyzed with and without scrubbing.** The left column shows the results analyzed without scrubbing; the right column shows the results of reanalysis with the bad frames removed. The first, second, and last row represent the discriminative gFCD, lFCD, and lrFCD, respectively.


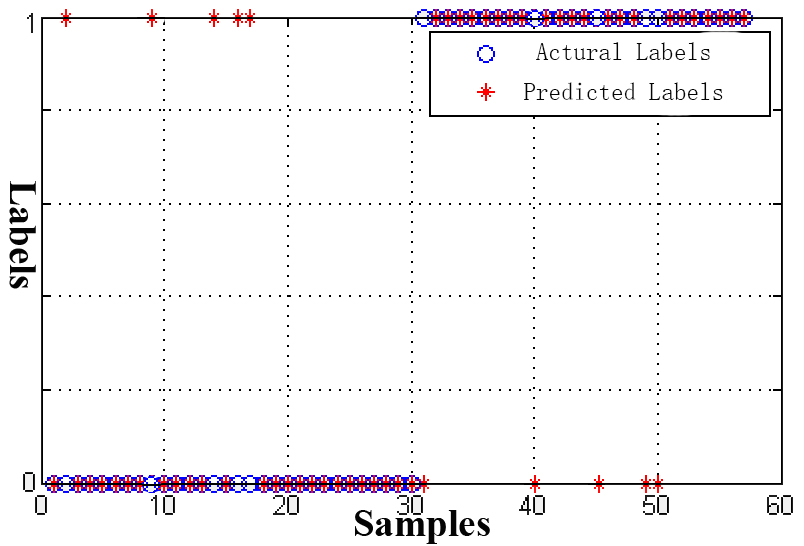


**Supplementary Figure 2. The classification performance of the SVC model based on the scrubbed fMRI data.** After removing the bad frames, the SVC model successfully identified IA subjects from HCs with a mean accuracy of 82.5%.


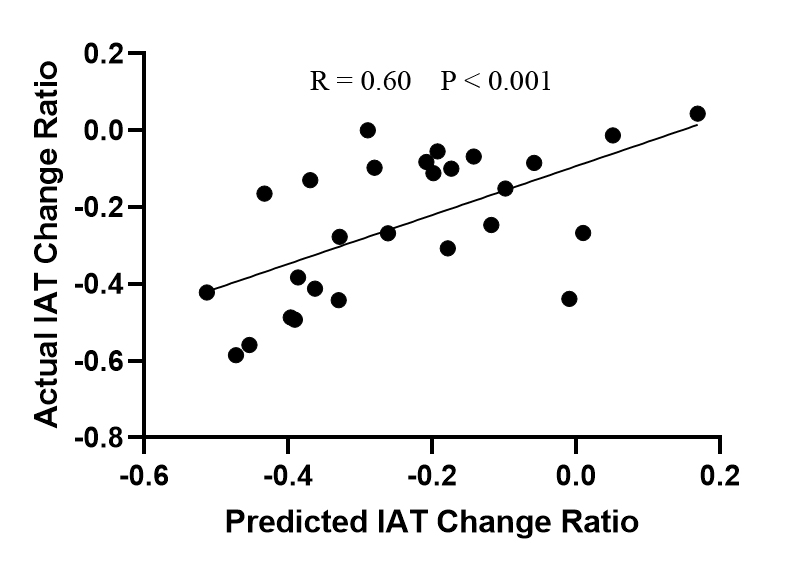


**Supplementary Figure 3. The regression performance of the SVR model based on the scrubbed fMRI data.** After scrubbing the bad frames, the SVR model predicted the IAT decrease with a correlation efficient of 0.60.
